# Expanding the application of non-invasive prenatal testing in the detection of foetal chromosomal copy number variations

**DOI:** 10.1186/s12920-021-01131-6

**Published:** 2021-12-11

**Authors:** Chaohong Wang, Junxiang Tang, Keting Tong, Daoqi Huang, Huayu Tu, Qingnan Li, Jiansheng Zhu

**Affiliations:** 1 Affiliated Maternity and Child Health Hospital of Anhui Medical University, Maternity and Child Health Hospital of Anhui Province, Hefei, China; 2 Beijing Genomics Institute, Beijing, China

**Keywords:** Non-invasive prenatal testing (NIPT), Chromosomal aneuploidy, Chromosomal microdeletion/microduplication, Copy number variation (CNV), Chromosomal microarray analysis (CMA)

## Abstract

**Purpose:**

The aim of this study was to assess the detection efficiency and clinical application value of non-invasive prenatal testing (NIPT) for foetal copy number variants (CNVs) in clinical samples from 39,002 prospective cases.

**Methods:**

A total of 39,002 pregnant women who received NIPT by next-generation sequencing (NGS) with a sequencing depth of 6 M reads in our centre from January 2018 to April 2020 were enrolled. Chromosomal microarray analysis (CMA) was further used to diagnose suspected chromosomal aneuploidies and chromosomal microdeletion/microduplication for consistency assessment.

**Results:**

A total of 473 pregnancies (1.213%) were positive for clinically significant foetal chromosome abnormalities by NIPT. This group comprised 99 trisomy 21 (T21, 0.254%), 30 trisomy 18 (T18, 0.077%), 25 trisomy 13 (T13, 0.064%), 155 sex chromosome aneuploidy (SCA, 0.398%), 69 rare trisomy (0.177%), and 95 microdeletion/microduplication syndrome (MMS, 0.244%) cases. Based on follow-up tests, the positive predictive values (PPVs) for the T21, T18, T13, SCA, rare trisomy, and MMS cases were calculated to be 88.89%, 53.33%, 20.00%, 40.22%, 4.88%, and 49.02%, respectively. In addition, the PPVs of CNVs of < 5 Mb, 5–10 Mb, and > 10 Mb were 54.55%, 38.46%, and 40.00%, respectively. Among the 95 cases with suspected CNVs, 25 were diagnosed as true positive and 26 cases as false positive; follow-up prenatal diagnosis by CMA was not performed for 44 cases. Moreover, among the 25 true positive cases, 10 were pathogenic, 3 were likely pathogenic, and 12 were of uncertain significance.

**Conclusion:**

NIPT is not only suitable for screening T21, T18, T13, and SCA but also has potential significance for CNV detection. As combined with ultrasound, extended NIPT is effective for screening MMS. However, NIPT should not be recommended for whole-chromosome aneuploidy screening.

## Introduction

Since the first detection of cell-free foetal DNA (cffDNA) in the plasma of pregnant women in 1997 [1], non-invasive prenatal testing (NIPT) detection for foetal chromosomal aneuploidy evaluation via extraction of cffDNA from maternal peripheral blood has been widely used in clinical practice in more than 60 countries. As an alternative screening method, NIPT is proven to have very high sensitivity and specificity for detecting common chromosomal aneuploidies, such as trisomy 21 (T21), trisomy 18 (T18), and trisomy 13 (T13), with low false positive and false negative rates [2–4]. Interestingly, recent studies have shown that in addition to T21, T18, and T13, NIPT is effective at detecting sex chromosome abnormalities (SCAs) [5, 6]. Indeed, the American College of Medical Genetics suggests that NIPT is the most sensitive method for prenatal screening of trisomy 21, 18, and 13.

Microdeletion/microduplication syndromes (MMS), which are due to copy number variations (CNVs), are another major inheritance factor that causes birth defects. CNVs are widespread in the human genome. The incidence of pathogenic CNVs in the normal population can reach 1.0–1.7%, which is much higher than the incidence of T21 (0.13–0.17%) [7]. Recently, the further development and expansion of NIPT have focused on MMS. However, the accuracy and reliability of MMS detection by NIPT is challenging because of the influence of some biological factors, such as a low content of cffDNA, maternal chromosomal abnormalities, and confined placental mosaicism [8]. Many attempts have been made to resolve these issues. Further sequencing or high-density single-nucleotide polymorphism-targeting methods have proven the feasibility of MMS detection by NIPT [9–11]. In China, Chen et al. [12] and Liang et al. [13] confirmed that NIPT is suitable not only for common chromosome aneuploidy detection but also for MMS detection. However, many problems and challenges in clinical practice remain. For example, the number of clinical samples remains insufficient, and MMS classification and follow-up are not ideal, which limits the large-scale application of MMS detection in NIPT.

This study aimed to assess the detection efficiency and clinical application value of NIPT for foetal copy number variants (CNVs) in clinical samples from 39,002 prospective cases, comprising the largest population study in East China. The findings of this study might help to improve diagnostic accuracy while reducing cost and improving maternal and prenatal health care.

## Methods

### Subjects

Within a 28-month period (January 2018 to April 2020), 39,002 pregnant women (age: 18–48 years old; gestational week: 12^+0^ ~ 26^+5^) in our centre were enrolled for this NIPT trial by NGS with a sequencing depth of 6 M reads. All patients underwent pretest counselling to inform them of the sample type, test method, test contents, test limitations, sensitivity and specificity of NIPT, etc. Ultrasound was required to determine the number of foetuses and to exclude structurally abnormal foetuses before testing. All high-risk pregnant women were advised to undergo amniocentesis and confirmatory prenatal diagnosis. We conducted a series of tests, such as foetal ultrasound and clinical examination of new-borns, for low-risk cases and patients with positive results who refused a prenatal diagnosis. All cases with a positive prenatal diagnosis were scheduled for genetic counselling. Among the 39,002 pregnant women, 7968 (20.43%) were older than 35 years old. In 832 (2.13%) of the women, abnormal single ultrasonic soft indexes, such as unilateral or bilateral choroid plexus cysts, ventricular bright spots, and slightly widened lateral ventricles, were detected. Critical thickening of the NT value was found in 237 cases (0.61%). In total, 5313 patients (13.62%) had a high risk of serological screening, 11,351 (29.10%) had a critical risk of serological screening, 74 (0.19%) had other causes, and 13,227 (33.91%) had no clinical indications but required NIPT detection with independent requirements.

### Indications for NIPT

The inclusion criteria were as follows: maternal serological screening critical-risk value (1/1000 ≤ T21 < 1/270, 1/1000 ≤ T18 < 1/350), maternal serological screening high-risk value (T21 ≥ 1/270, T18 ≥ 1/350), contraindications of prenatal diagnosis, missed serological screening or requested NIPT, advanced maternal age (35 years or older at the expected due date), abnormal single ultrasonic soft indexes such as unilateral or bilateral choroid plexus cyst, ventricular bright spot, slightly widened lateral ventricle, critical thickening of the NT value (2.0 ≤ NT ≤ 3.0), IVF, or twin pregnancy.

Exclusion criteria for NIPT were as follows: not in gestational week 12 + 0 ~ 26 + 5, chromosome abnormality in one of the parents, ultrasonography showing structurally abnormal foetuses, family history of genetic diseases or high-risk of genetic diseases, malignant tumour during pregnancy, allogeneic blood received within one year, exogenous DNA introduced within 4 weeks, and transplantation and stem cell therapy, among others.

Chromosomal aneuploidies suspected by NIPT were confirmed by karyotype analysis. In pregnancies with copy number variants shown by NIPT, we suggest the application of CMA at prenatal diagnosis. Informed written consent was obtained from all participants for the use of their peripheral blood and amniotic fluid samples for the current analyses.

### Collection and treatment of blood samples

Maternal peripheral blood samples (5 mL) were collected in EDTA tubes, fully mixed, and stored temporarily at 4 ℃. Samples were excluded if haemolysis or storage occurred beyond eight hours before plasma separation. The blood sample was centrifuged at 4 ℃ and 1600×*g* for 10 min, and the plasma was collected carefully and dispensed into 2.0-mL Eppendorf tubes. The plasma was centrifuged again at 4 ℃ and 16,000×*g* for another 10 min. The upper plasma was carefully divided into new 2.0-mL Eppendorf tubes, with each tube containing approximately 600uL plasma, which was stored at −80 ℃. Repeated freezing and thawing before the experiment, as previously reported, were avoided [14].

### NIPT with the BGISEQ-500 sequencing platform

DNA extraction, library construction, and sequencing were performed according to the standard protocol of Human Molecular Genetics Guidelines at Anhui Maternal and Child Health Care Hospital. Maternal plasma (200 μL) was used for cell-free foetal DNA extraction with a BGISP-300 (BGI, Shenzhen, China) and Nucleic Acid Extraction Kit (BGI, Shenzhen, China). After DNA extraction, the DNA was subjected to end repair by end repair enzymes under the following conditions: 37 °C for 10 min and 65 °C for 15 min, followed by adaptor ligation at 23 °C for 20 min with the label adaptor and ligase. After end repair and adaptor ligation, PCR was used to amplify the DNA to the desired concentration with the following cycle conditions: 98 °C for 2 min, followed by 12 cycles at 98 °C for 15 s, 56 °C for 15 s, and 72 °C for 30 s and a final extension at 72 °C for 5 min. The DNA amplification products were quantified using a Qubit® 2.0 (Life Tech, Invitrogen, USA) and QubitTM dsDNA HS Assay Kits (Life Tech, Invitrogen, USA); a concentration ≥ 2 ng/μL was regarded as a qualified standard. The volume was calculated according to the concentration of each sample, and each sample of the same mass was mixed by pooling. Double-stranded DNA was thermally denatured after pooling, and cyclic buffer and ligase were added for a cyclization reaction. The DNA circles were used to prepare DNBs by rolling circle replication (RCR). The concentration of DNBs was quantified by a Qubit® 2.0 using QubitTM ssDNA Assay Kits (Life Tech, Invitrogen, USA), and DNB concentrations in the range of 8–40 ng/μL were considered appropriate. The DNBs were loaded onto chips and sequenced using the BGISEQ-500 sequencing platform [14] (BGI, Shenzhen, China). Any sample that failed to meet the quality control criteria was reported as detection failure by NIPT.

The sequence based on NGS was compared with the reference sequence map of the human genome, and the percentage of each chromosome was calculated with Illumina Sequencing Analysis Viewer1.9.1 software. Z and T values were used to evaluate the actual disease situation of the samples, as previously reported [14]. Interventional prenatal diagnosis was recommended for high-risk pregnant women with NIPT, and the pregnancy outcomes of all cases were followed up.

### Karyotype analysis

Informed written consent was obtained from all participants for the use of their peripheral blood and amniotic fluid samples for the current analyses, and the tests were performed with patient agreement. Amniocentesis and confirmatory prenatal diagnosis were recommended for all high-risk pregnancies. Karyotypes were analysed according to the standard of ISCN (2016) through the process of standardized cell culture, filmmaking, and G-banding.

### Chromosomal microarray analysis (CMA)

Amniotic fluid samples were centrifuged at 4 ℃ and 1000×*g* for 10 min; the cell pellet was retained for extraction of DNA. A 4-μL aliquot of DNA sample with absorbance (A260/280 nm) at 1.7–1.9 was used, and chromosomal microarrays were detected according to the instructions of Affymetrix CytoScan 750 K arrays (Thermo Fisher, MA, USA). Data were collected by the GeneChip™ Scanner 3000 system, and the results were analysed by Chromosome Analysis Suite software.

## Results

### Overall NIPT screening results for 39,002 cases

In 412 cases (1.056%, 412 per 39,002), blood needed to be drawn again due to a variety of reasons, such as severe haemolysis and coagulation, large fluctuation of the data for chr21, chr18 and chr13, large fluctuation of the data for other chromosomes, SCA, low content of cffDNA (< 5%), and high DNA concentration. Among these 412 cases, 384 cases (93.204%, 384 out of 412) were effectively detected after drawing blood again; however, 28 cases (6.796%, 28 out of 412) were not, suggesting that the detection failure rate of NIPT was 0.072% (28 out of 39,002).

**Fig. 1:**
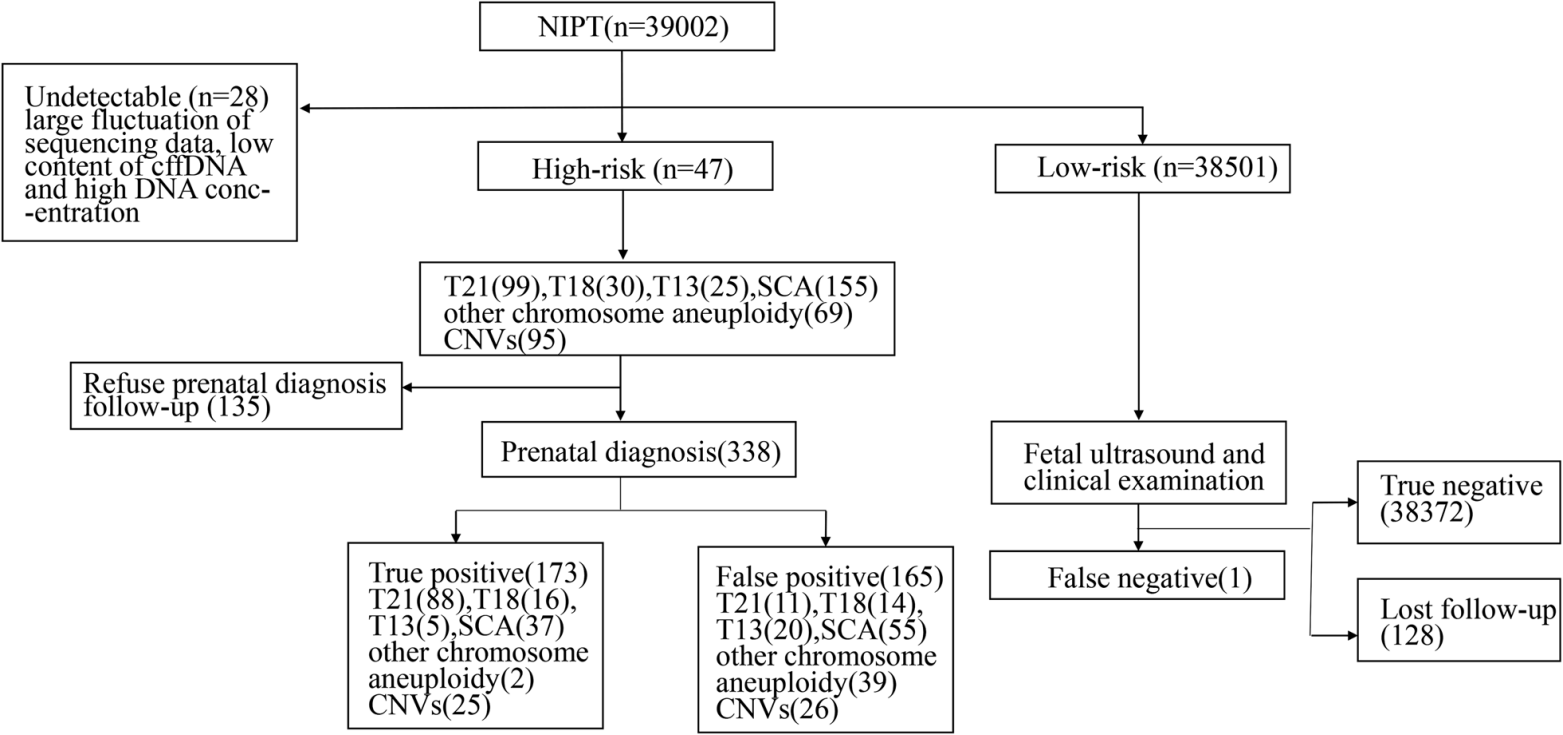
Flowchart of non-invasive prenatal test (NIPT) results

Among 38,974 cases with effective detection, 473 cases were high-risk, comprising 99 trisomy 21 (T21, 0.254%), 30 trisomy 18 (T18, 0.077%), 25 trisomy 13 (T13, 0.064%), 155 sex chromosomal aneuploidies (SCA, 0.398%), 69 rare trisomy (0.177%), and 95 microdeletion/microduplication syndrome (MMS, 0.244%) cases. Of these, 338 patients (71.459%, 338 out of 473) agreed to follow-up prenatal diagnoses, whereas 135 (28.541%, 135 out of 473) refused. For low-risk cases and patients with positive results who refused to receive a prenatal diagnosis, we conducted a series of tests, such as foetal ultrasound and clinical examination of new-borns; however, 128 patients were lost to follow-up. After follow-up, we found 1 case of T21 among the NIPT-negative results. The flowchart of the study is illustrated in Fig. 1.

### Detection efficiency of T21, T18, and T13 in NIPT

To evaluate the clinical efficacy of NIPT in this study, we focused on the detection efficiency of T21, T18, and T13 for overall evaluation. There were 154 patients with a high risk of T21, T18, and T13 among 38,974 patients, and all were followed up by amniocentesis and karyotype analysis. As shown in Table 1, the positive predictive values (PPVs) of T21, T18, and T13 were 88.89% (88 out of 99), 53.33% (16 out of 30), and 20.00% (5 out of 25), respectively; sensitivities were 98.88%, 100.00%, and 100.00% and specificities 99.97%, 99.96%, and 99.95%, respectively. However, because one case of T21 was missed, which was found during follow-up, the negative predictive value (NPV) for T21 was 99.99% (Table 1).

**Table 1:** The detection efficiency of T21, T18, and T13 in NIPT

NIPT	TP	FP	PPV (%)	Sensitivity (%)	TN	FN	NPV (%)	Specificity (%)
T21	88	11	88.89	98.88	38,874	1	99.99	99.97
T18	16	14	53.33	100	38,944	0	100	99.96
T13	5	20	20	100	38,949	0	100	99.95

### Detection efficiency of NIPT in screening other chromosome aneuploidies

After evaluating T21, T18, and T13, we sought to evaluate the occurrence of other chromosomal abnormalities and CNVs. First, we examined SCAs and found the following: (1) 92 patients agreed to follow-up prenatal diagnosis by karyotype analysis, and 37 cases were true positive (PPV: 40.22%); (2) 71 patients had a high risk of 45,X, but only 40 were followed up by amniocentesis and karyotype analysis, and 7 cases were true positive (PPV: 17.50%); (3) 39 patients had a high risk of 47,XXY, but only 23 were followed up by amniocentesis and karyotype analysis, and 13 cases were true positive (PPV: 56.52%); (4) 30 patients had a high risk of 47,XXX, but only 18 were followed up by amniocentesis and karyotype analysis, and 10 cases were true positive (PPV: 55.56%); (5) 15 patients had a high risk of 47,XYY, but only 11 were followed up by amniocentesis and karyotype analysis, and 7 cases were true positive (PPV: 63.64%). Second, the detection efficiency of NIPT for other chromosomal abnormalities was relatively low. Among 41 cases followed up by amniocentesis and karyotype analysis, only 2 were true positive (PPV: 4.88%). The highest incidence rate of other chromosomal abnormalities in this study was for T7 and T16 (13 cases); T8 was found in 12 cases. Unfortunately, in addition to T2, the PPVs of other chromosomal abnormalities, including T7, T8, T16, monosomic 18 (M18), T15, T2, T19, monosomic 21 (M21), T3, T5, T6, T10, and T14, were 0% (Table 2).

**Table 2:** The detection efficiency of SCA, other chromosome aneuploidy, and CNVs in NIPT

NIPT	Positive	TP	FP	Unverified	PPV (%)
SCA	155	37	55	63	40.22
45,X	71	7	33	31	17.50
47,XXY	39	13	10	16	56.52
47,XXX	30	10	8	12	55.56
47,XYY	15	7	4	4	64.64
Other chromosome aneuploidy	69	3	38	28	7.32
T7	13	0	8 (likely placental mosaicism)	5	Not estimable
T8	12	0	7 (likely placental mosaicism)	5	Not estimable
T16	13	0	7 (likely placental mosaicism)	7	Not estimable
M18	4	0	4	0	0.00
T15	6	0	3	3	0.00
T2	5	2	1	2	66.67
T9/M21	5	0	4 (2/2)	1	0.00
T3/T5/T6/T10/T14	11	0	5 (1/1/1/1/1)	6	0.00
CNV	95	25	26	44	49.02
CNV < 5 Mb	42	18	15	9	54.55
CNV 5–10 Mb	25	5	8	12	38.46
CNV > 10 Mb	28	2	5	21	40.00

### Detection efficiency of NIPT in CNV screening

**Fig. 2:**
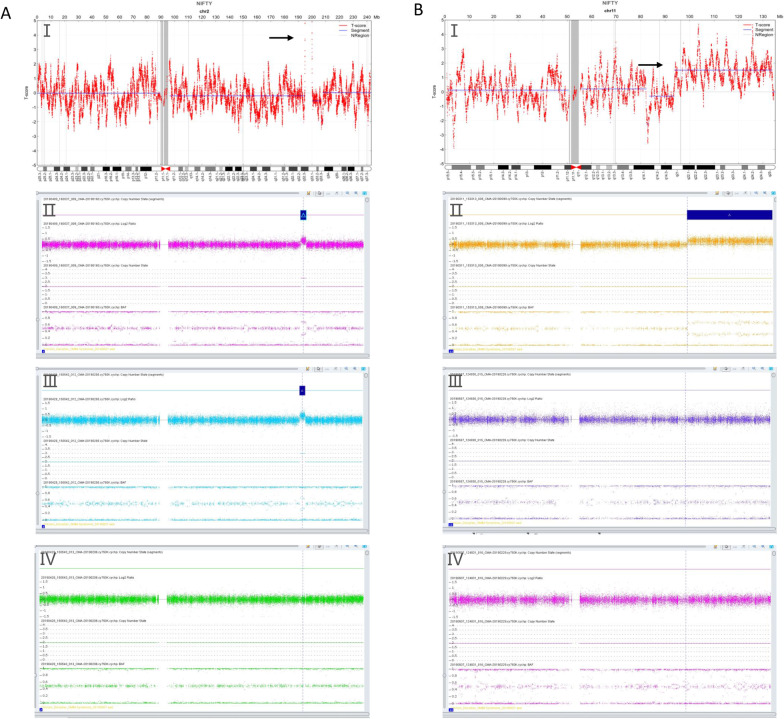
Comparison of NIPT and CMA in detecting duplications. **AI** NIPT of peripheral blood from the pregnant woman. The genomic position is shown on the x-axis and the count-based *T*-score is shown on the y-axis, arrow indicates the sign of duplication region detected by NIPT: 5.29 Mb at chromosome 2q32.3q33.1. Because the gain in copy number go off scale, it could be speculated that this duplication is maternal in origin. **AII–AIII.** CMA as indicated by copy number state using the Affymetrix Cytoscan 750 K.The chromosome duplication region of the fetus and pregnant women from 195,365,474 to 199,646,763 bp at chromosome 2 is indicated by bule box. The upper axis indicates the log2 ratio, where 2 illustrates the ratio of each SNP compared to diploid individuals in chromosome (a log2 ratio of 0 means an actual ratio of 1). Additionally, the lower abscissa axis indicates the value of B allele frequency (BAF). BAF may have three values in a diploid individual. While the BAF with four values and the increased log2 ratio indicated duplication regions. **AIV** The CMA result of the pregnant women’s husband. **BI** NIPT of peripheral blood from a pregnant woman shows gain in copy number at chromosome 11q21q25 with 40.09 Mb, arrow indicates the sign of duplication region detected by NIPT. **BII** The CMA result of the fetus shows the chromosome duplication region from 99,364,342 to 134,937,416 bp at chromosome 11q22.1q25 by bule box. **BIII–BIV**. The CMA results of the parents revealed a de novo 35.57 Mb duplication at 11q22.1q25 (chromosome position: 99,364,342–134,937,416)

MMS, which is due to copy number variations (CNVs), is another major inheritance factor that causes birth defects, and we thus analysed CNVs in NIPT. Among the 51 cases for which CMA and/or karyotype analysis could subsequently achieve prenatal diagnosis, 25 were true positive (PPV: 49.02%) (Table 2 and Fig. 2). Then, we subdivided these 51 cases according to the CNV fragment size. The PPVs of < 5 Mb, 5–10 Mb, and > 10 Mb CNVs were 54.55%, 38.46%, and 40.00%, respectively (Table 2). To further examine the distribution of true positive CNVs, we classified them by chromosome. Interestingly, the PPVs of CNVs in different chromosomes showed obvious differences. For example, the PPVs of CNVs on chr3 were 100.00% and on chr5 were 0%(Table 3). Moreover, through the combination of ACMG standardized analysis and follow-up visits, among the 25 true positive cases, 10 were pathogenic and 3 likely pathogenic; 12 cases were of uncertain significance (Table 4).

**Table 3:** The distribution of true positive CNVs in different chromosomes

Chr	CNVs length			CNV gain or loss		CCNV		
	< 5	Within 5–10 Mb	> 10	CNV	CNV	NIPT	NIPT true	NIPT false
	Mb		Mb	Gain	Loss	positive	positive	positive
Chr1	3	0	0	2	1	3	1	2
Chr2	3	1	0	2	2	4	3	1
Chr3	2	1	0	3	0	3	3	0
Chr4	1	0	0	0	1	1	1	0
Chr5	3	1	2	0	6	6	0	6
Chr6	2	1	0	2	1	3	1	2
Chr7	0	1	0	0	1	1	0	1
Chr8	2	1	0	1	2	3	2	1
Chr9	0	1	0	1	0	1	0	1
Chr10	1	1	0	0	2	2	1	1
Chr11	1	0	1	2	0	2	2	0
Chr12	1	0	0	0	1	1	1	0
Chr13	2	1	0	1	2	3	1	2
Chr14	0	0	0	0	0	0	0	0
Chr15	1	2	0	1	2	3	2	1
Chr16	3	0	0	2	1	3	2	1
Chr17	2	0	0	1	1	2	1	1
Chr18	4	1	1	3	3	6	4	2
Chr19	0	0	0	0	0	0	0	0
Chr20	1	0	1	1	1	2	0	2
Chr21	0	0	0	0	0	0	0	0
Chr22	1	1	0	1	1	2	0	2
Total	33	13	5	23	28	51	25	26

**Table 4:** Classification of true positive CNV and pregnancy outcome

Patients	NIPT	CMA	CNV	Inheritance	Pregnancy outcome	Gestation at the time of therapeutic abortion
Case 1	8p23.2p23.3, deletion, 5.2 M	8p23.2p23.3, deletion, 6.0 M	Pathogenic	de novo	Therapeutic abortion	23^+2^ W
Case 2	12p12.1p12.2, deletion, 4.2 M	12p12.1p12., deletion, 4.13 M	Pathogenic	not determined	Therapeutic abortion	21^+5^W
Case 3	18q12.3, duplication, 2.2 M	18q12.3, duplication, 1.51 M	VOUS	Not determined	Delivery, normal	
Case 4	15q13.2q13.3,deletion, 2.1 M	15q13.2q13.3, deletion, 1.34 M	Pathogenic	de novo	Therapeutic abortion	23^+4^ W
Case 5	11p14.2p14.3, duplication, 3.85 M	11p14.2p14.3, duplication, 3.59 M	VOUS	Inherited from the father	Delivery, normal	
Case 6	11q22.1q25, duplication, 40.09 M	11q22.1q25, duplication, 35.57 M	Pathogenic	de novo	Head deformity (ultrasound), therapeutic abortion	22^+1^ W
Case 7	16p13.11, duplication, 2.5 M	16p13.11, duplication, 1.64 M	Likely pathogenic (nonpenetrance)	Not determined	Unknown	
Case 8	10q21.1, deletion, 2.1 M	10q21.1, deletion, 2.13 M	VOUS	Not determined	Delivery, normal	
Case 9	2q32.3q33.1, duplication, 5.29 M	2q32.3q33.1, duplication, 4.28 M	VOUS	Inherited from the mother	Delivery, normal	
Case 10	18q22.3q23, duplication, 5.45 M	18q22.3q23, duplication, 6.04 M	VOUS	Inherited from the mother	Head deformity (ultrasound), therapeutic abortion	26^+6^ W
Case 11	4p14p15.1, deletion, 3.6 M	4p14p15.1, deletion, 3.32 M	VOUS	Not determined	Delivery, normal	
Case 12	13q33.1q34, deletion, 9.4 M	13q33.1q34, deletion, 9.22 M	Pathogenic	de novo	Craniofacial malformation (ultrasound), therapeutic abortion	25^+1^ W
Case 13	1q21.1q21.2, duplication, 3.0 M	1q21.1q21.2, duplication, 1.71 M	Pathogenic (incomplete penetrance)	Inherited from the mother	Therapeutic abortion	24^+6^ W
Case 14	3q26.33q27.1, duplication, 3.35 M	3q26.33q27.1, duplication, 3.29 M	VOUS	Not determined	Delivery, normal	
Case 15	15q11.2q13.1, duplication, 6 M	15q11.2q13.1, duplication, 5.24 M	Pathogenic	Not determined	Therapeutic abortion	
Case 16	18q12.3q22.3, duplication, 28.6 M	18q12.3q22.3, duplication, 30.59 M	Pathogenic	Not determined	Head deformity and cheilopalatognathus (ultrasound), therapeutic abortion	23^+5^ W
Case 17	16p13.11p12.3, duplication, 3.6 M	16p13.11p12.3, duplication, 2.67 M	Likely pathogenic (nonpenetrance)	not determined	Delivery, normal	
Case 18	3p26.3, duplication, 2.5 M	3p26.3, duplication, 2.21 M	VOUS	Inherited from the mother	Delivery, normal	
Case 19	2q12.1q12.3, duplication, 2.5 M	2q12.1–12.3, duplication, 2.51 M	VOUS	Not determined	Delivery, normal	
Case 20	17p12, deletion, 2.5 M	17p12, deletion, 1.42 M	Pathogenic	Inherited from the mother	Delivery, normal	
Case 21	6p12.1p12.3, duplication, 4.6 M	6p12.1p12.3, duplication, 4.36 M	VOUS	Inherited from the mother	Delivery, normal	
Case 22	18q12.2, deletion, 2.5 M	18q12.2, deletion, 1.06 M	VOUS	Not determined	Therapeutic abortion	23^+3^ W
Case 23	2q13, deletion, 2 M	2q13, deletion, 2.12 M	Likely pathogenic (nonpenetrance)	Inherited from the mother	Delivery, normal	
Case 24	8 p21.3p22, deletion, 5.21	8p21.3p2, deletion, 3.81 M	VOUS	Not determined	Delivery, normal	
Case 25	3q26.32q28, duplication, 9.61 M	3q26.32q28, duplication, 9.61 M	Pathogenic	de novo	Facial and cardiac abnormalities (ultrasound), therapeutic abortion	26^+1^ W

## Discussion

As an alternative screening method, NIPT has been shown to have very high sensitivity and specificity for detecting common chromosomal aneuploidies such as T21, T18, and T13, with low false positive and false negative rates. However, screening of other chromosome aneuploidies and CNVs is still controversial [15, 16]. In 2016, the ACMG suggested that NIPT is only suitable for screening common chromosomal aneuploidy abnormalities and that positive results need to be verified by invasive prenatal diagnosis [17]. In addition to chromosomal aneuploidies, MMS, which is due to copy number variations (CNVs), is another major inheritance factor that causes birth defects. Indeed, the incidence of MMS in the normal population can reach 1.00%-1.70%, which is even higher than the incidence of T21 (0.13–0.17%). In practice, CMA is a powerful tool for detecting MMS and has been recommended as a primary diagnostic tool for some definite syndromes [18]. Nevertheless, CMA requires invasive sampling, causing unnecessary risks [19], such as miscarriage, preterm birth, and intrauterine infection, and it may thus be rejected by some women. Based on the excellent performance of NIPT in the detection of CNVs, an increasing number of guidelines suggest that it should be applied in the screening of CNVs [20]. Thus, the aim of this study was to assess the detection efficiency and clinical application value of NIPT for CNVs in clinical samples from 39,002 prospective cases and to accumulate experience for subsequent large-scale application.

It has been reported that the PPV range of NIPT is 65–94% for T21, 47–85% for T18, and 12–62% for T13 [21–23]. In this study, the PPVs of T21, T18 and T13 were 88.89%, 53.33% and 20.00%, respectively, consistent with reported results. Of the 38,974 of 39,002 cases that could be detected by NIPT, cases of T18 and T13 were correctly identified, and the NPVs were both 100%. Only one case of T21 was missed, and the NPV was 99.99%, suggesting that NIPT screening for T21, T18, and T13 displayed excellent accuracy and reliability in this study. In the case of the T21 missed diagnosis, NIPT data showed a low risk of T21. The karyotype using the amniotic fluid sample was 47,XY, + 21[67]/46,XY[16]. After induction of labour, a CNV-seq test of placental tissue showed T21 mosaicism, implying that mosaicism was the main cause of the false negative in this case.

In addition, we analysed the detection efficiency of SCA in NIPT and found a PPV of 40.22% for all SCAs. The PPVs of 45,X, 47,XXY, 47,XXX, and 47,XYY were 17.50%, 56.52%, 55.56%, and 63.64%, respectively. NIPT had relatively high PPVs for 47,XXY, 47,XXX, and 47,XYY and a low PPV for 45,X. The following were considered as possible explanations: (1) the high GC content of the X chromosome led to poor amplification efficiency; (2) X and Y chromosome sequences are highly homologous and prone to deviation in sequence comparison; and (3) random inactivation of the X chromosome. In conclusion, NIPT is suitable for the detection of SCA, but results for 45,X should be treated with caution.

There were 69 cases of other chromosome aneuploidy abnormalities in this study, including 2 true positive cases, 39 false positive cases, and 28 undetermined cases. The PPV of other chromosome aneuploidy abnormalities was 4.88%. Two true positive cases showed a duplication on chr2. The amniotic fluid karyotype in one case was mos47,XX, + 2[11]/46,XX[47]; the CMA result showed 60–70% chromosomal duplication mosaicism on chr2, with no abnormality on ultrasound. The amniotic fluid karyotype for one case was 46,XX, and CMA showed chromosomal duplication mosaicism on chr2 of ~ 30%, with no ultrasound abnormality. After careful selection, 2 pregnant women chose to continue the pregnancy; these 2 children are approximately 1 year old, with normal intelligence, growth and development. Overall, the trophoblast cells detected by NIPT and exfoliated cells detected in amniotic fluid do not completely represent the condition of the foetus. In clinical practice, especially in mosaicism, ultrasound needs to be added for comprehensive evaluation. Among the cases, T7 was most frequent, with 8 cases in total, followed by T8 and T16, though no abnormality was found by amniotic fluid verification or ultrasound. However, Qi et al. [24–26] suggested that T7, T8, and T16 cases should almost certainly be interpreted as placental mosaicism and as confined placental mosaicism if accompanied by normal amniocentesis data. The possible causes of false positives in NIPT are as follows. (1) The accuracy of NIPT results depend not only on the detection ability but also on the incidence of disease [27]. As other chromosomal aneuploidies are rare, the PPVs of other chromosomal aneuploidies screened by NIPT were not high, which may be the reason why the PPVs of T13 and T18 were significantly lower than that of T21. (2) Chromosomal abnormalities only occurring in the placenta and not in the foetus are called confined placental mosaicism (CPM) [28]. The foetal free DNA detected by NIPT mainly originates from placental trophoblast cells; thus, CPM is another main reason for false positive cases, with an incidence of approximately 1–2% [8]. (3) The false positive rate of NIPT might be due to a trisomy rescue mechanism, i.e., uniparental disomy (UPD) [29]. In this study, one case showed a duplication on chr16. The karyotype based on amniotic fluid was 46,XY, and the CMA results showed 5.91 Mb loss of heterozygosity in the region of 16p13.3, which did not rule out the possibility of UPD on chr16. It is clear that chr.6, 7, 11, 14, 15, and 20 are involved in imprinting syndrome. Considering that there is no known imprinted gene on chr16, the pregnant woman finally chose to continue the pregnancy after combining high-level ultrasound monitoring. In conclusion, other chromosomal aneuploidy abnormalities indicated by NIPT usually had favourable pregnancy outcomes in this study. In general, NIPT should not be recommended for screening of other chromosomal aneuploidies, and invasive testing should be used cautiously.

Because of the high incidence of MMS (1.00–1.70%), Liang et al. [13] suggested that extended NIPT combined with ultrasound can be used as an independent system for foetal MMS screening. Overall, the accuracy of detecting MMS by NIPT may be affected by many factors, such as the cffDNA content, interference of maternal CNVs, and interference of foetal placenta mosaicism. At present, the accuracy of detection is effectively improved by GC correction comparison and sliding window analysis of data. MMS in this study also had an excellent PPV (49.02%); the PPVs of CNVs with < 5 Mb, 5–10 Mb, and > 10 Mb were 54.55%, 38.46%, and 40.00%, respectively. According to ACMG standards, CNVs can be divided into five categories: pathogenic, likely pathogenic, uncertain significance, likely benign, and benign. Among the 25 true positive cases, 10 were pathogenic, 3 were likely pathogenic, and 12 were of uncertain significance. The condition of pathogenic CNVs in 10 cases may be summarized as follows. (1) There were 4 cases with abnormal ultrasound results, and the women chose therapeutic abortion. (2) There were 4 cases without abnormal ultrasound results; considering that pathogenicity was mostly related to developmental retardation, mental retardation, language retardation, attention deficit hyperactivity disorder or autistic behaviour, the pregnant women also chose therapeutic abortion. (3) There was 1 case of a 1.42-Mb deletion of 17p12, the pathogenicity of which is related to hereditary pressure susceptible peripheral neuropathy. CMA of the parents confirmed that it was inherited from the mother, and no abnormality was found on ultrasound. After careful consideration, the woman chose to continue the pregnancy and delivered a healthy baby. (4) There was 1 case with a 1.71-Mb duplication of 1q21.1q21.2, which is associated with 1q21.1 microduplication syndrome and mainly manifests as mild to moderate mental retardation, autism, attention-deficit hyperactivity disorder, giant, etc. The penetrance rate of this syndrome is approximately 29.1%. CMA of the parents confirmed that it was inherited from the mother, and no abnormality was found on ultrasound. After careful consideration, the woman chose therapeutic abortion given the 29% risk of penetrance. The condition of likely pathogenic CNVs in 3 cases may be summarized as follows. (1) One patient refused to be followed up, and the pregnancy outcome was unknown. (2) There was 1 case of a 2.67-Mb duplication of 16p13.11p12.3, which is a susceptible area for neurocognitive diseases. An individual carrying this duplication might manifest mental retardation, developmental retardation, autism, attention deficit hyperactivity disorder, etc. The penetrance rate of this syndrome is approximately 5–10%. Considering the low penetrance rate and normal ultrasound result, the woman chose to continue the pregnancy, and a healthy baby was born. (3) There was 1 case with a 2.12 Mb deletion of 2q13. According to CLINGEN, the deletion score of this region is 2. Individuals with this deletion possibly manifest mental retardation, autism, attention deficit hyperactivity disorder, congenital heart abnormality, mild facial abnormalities, etc. CMA of the parents confirmed that it was inherited from the mother. The woman chose to continue the pregnancy, and a healthy baby was born. CNVs of uncertain significance were identified in 12 cases, as follows. (1) Eleven cases without abnormal ultrasound results. After careful consideration, 10 patients chose to continue the pregnancy, and healthy babies were born; however, 1 woman chose induction of labour. (2) There was 1 case with a 6.04-Mb duplication of 18q22.3q23. CMA of the parents confirmed that it was inherited from the mother. The woman chose therapeutic abortion because of cranial abnormalities on ultrasound. Thus, among the 25 true positive cases, 13 had good pregnancy outcomes, suggesting that (1) the pathogenicity of CNV should be determined, (2) the detection scope should be limited to definite chromosome microdeletion and microduplication syndrome and (3) comprehensive analysis of ultrasound and medical history should be combined to reduce unnecessary invasive diagnostic measures.

## Conclusions

This is a systematic report of NIPT for foetal CNV detection using a large sample size. NIPT based on NGS is a potential method for foetal CNV detection, especially with further investigation about a large number of clinical samples and long-term detailed clinical followed-up. At present, extended NIPT combined with ultrasound is effective for screening of MMS, but NIPT should not be recommended for whole-chromosome aneuploidy screening.

## Data Availability

The datasets used or analysed during the current study are available from the corresponding author on reasonable request.
